# Drug free remission after steroid-dependent disappearance of lymphoproliferative disorder in rheumatoid arthritis patient treated with TNF-alpha blockade: case study

**DOI:** 10.1186/s40064-015-0798-9

**Published:** 2015-02-01

**Authors:** Kosaku Oda, Mutsuko Minata

**Affiliations:** Department of Orthopedic Surgery, Takatsuki Red Cross Hospital, 1-1-1 Abuno, Takatsuki, Osaka 569-1045 Japan; Department of Neurological Surgery, Ohio State University, 400 W Wiseman Hall 12th Ave, Columbus, Ohio 43210 USA

**Keywords:** Rheumatoid arthritis, Complete remission, Lymphoproliferative disorder, Tumor necrosis factor alpha inhibitor

## Abstract

**Introduction:**

TNF-α inhibitors plus MTX appear to have benefit in the longer-term reduction of RA. Boolean long-term remission under drug-free conditions is rare. The therapeutic mechanism and the factor of predicting response have not been clarified yet.

**Case description:**

A 24-year-old female rheumatoid arthritis (RA) patient, who once attained complete remission (CR) with the combination therapy with tumor necrosis factor alpha (TNF-alpha) inhibitor adalimumab (ADA) and methotrexate (MTX), showed the occurrence of Epstain- Barr virus (EBV)-associated lymphoproliferative disorder (LPD). Pulse treatment with methylprednisolone after the termination of anti TNF-α therapy resulted in the remission of EBV-associated LPD. The administration of prednisolone (PSL) was tapered off after the improvement of clinical symptoms and laboratory data. The patients achieved drug-free 12 months after urgent hospitalization and delivered healthy baby 2 years after hospital discharge. She has been complete drug-free Boolean remission for 5 years.

**Discussion and evaluation:**

The purpose of this brief case is report that we experienced the remission of LPD after CR with combined therapy with ADA and MTX. We believe this case report will be one of the paths for unveiling the pathogenesis and improving the treatment for RA.

**Conclusions:**

We believe this case report will be one of the paths for unveiling the pathogenesis and improving the treatment for RA.

## Background

Tumor necrosis factor-alpha (TNF-α) inhibitors have revolutionized the management of RA and other chronic inflammatory diseases. TNF-α inhibitors plus MTX appears to have benefit in the longer-term reduction of RA signs/symptoms in MTX-resistant patients, with no unexpected safety concerns. Nonetheless, Boolean defined disease remission occurred in 5.0-10.1% of RA, and long-term remission under drug-free conditions is rare (Shahouri et al. 2011). Moreover, these drugs have the increased risk of opportunistic infections and lymphoproliferative disorder (LPD) (Wolfe and Michaud 2004). The therapeutic mechanism and the factor of predicting response have not been clarified yet. Here we reported an RA patient who developed EBV-associated LPD in immediately after achieving complete remission (CR) by combination of low doses of MTX and TNF-α inhibitor adalimumab (ADA).

## Case description

**Figure 1:**
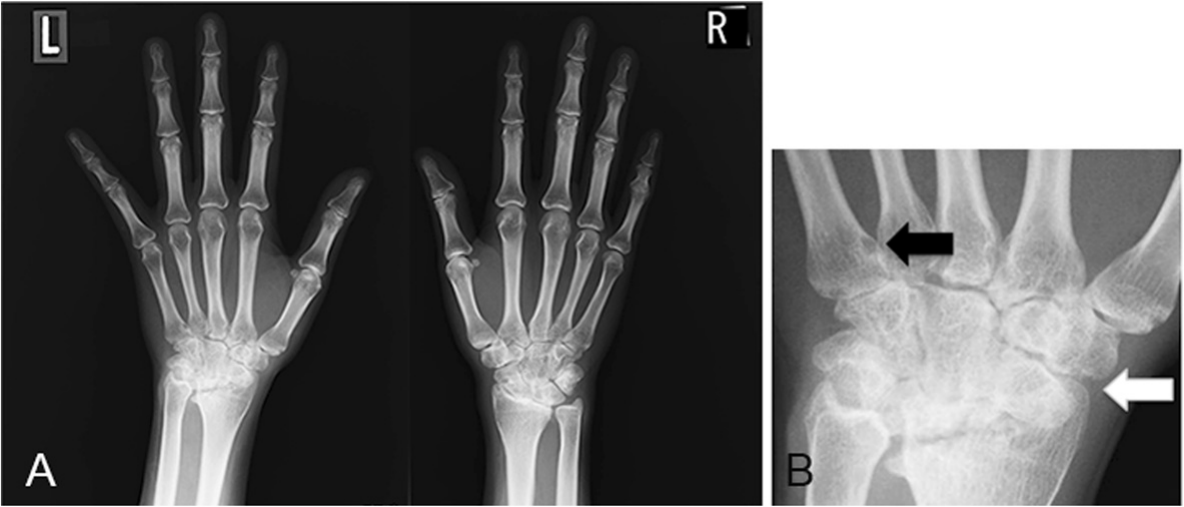
**X-ray of bilateral fingers and wrists at the diagnosis: A)** **erosions with deformity of the carpal bones in the hands,** **B)** **Left wrist demonstrating bone erosion (black arrow) and joint narrowing partially ankylosis and subluxation of the carpal bones (white arrow).**

**Figure 2:**
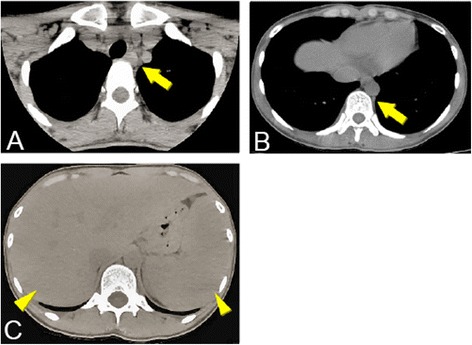
**Computed tomography (CT) from cervical to abdomen: A)** **and** **B)** **cervical lymph nodes swelling (** ***arrows*** **),** **C)** **hepatosplenomegary (** ***arrow heads*** **).**

**Figure 3:**
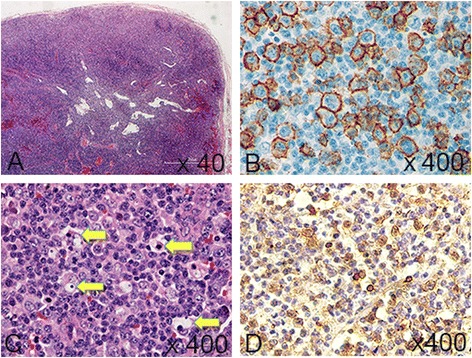
**Immunohistochemistry of inguinal lymph node: A)** **Hematoxylin and eosin stain (H&E stain) (** **×** **40 magnification),** **B)** **Immunostaining shows positive with anti-CD 20 antibody (** **×** **400 magnification),** **C)** **H&E staining in high power field shows proliferations of the full range of diffuse lymphatic cells and phagocytic macrophages included condensed small nuclear cells (** ***arrows)*** **suggested the presence of apoptotic cells (** **×** **400 magnification),** **D)** **Lymphatic cells are frequently positive with cleaved caspase 3 (** **×** **400 magnification).**

**Figure 4:**
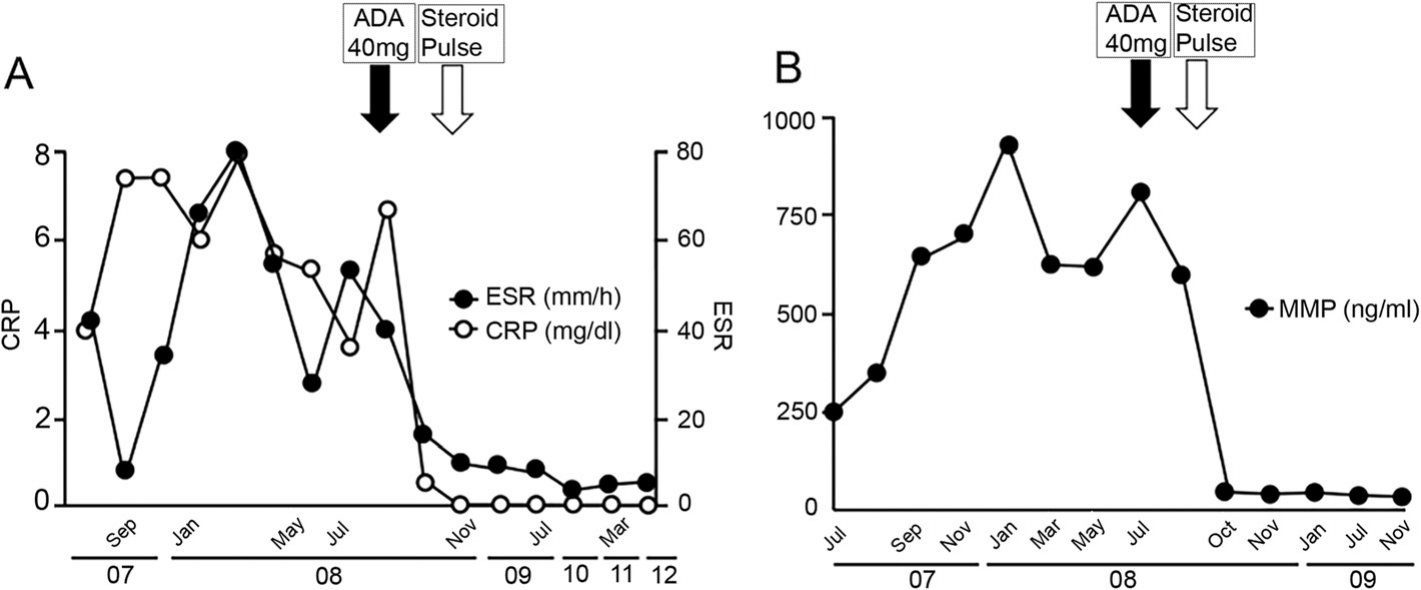
**Changes in inflammation and disease activity: A)** **CRP & ESR,** **B)** **MMP-3, Clinical course and the treatment showed that the inflammation and disease activity were improved after terminating of the MTX and ADA and administrating the steroid pulse treatment.**

A 24-year-old Japanese woman with one year history of active rheumatoid arthritis (RA) who complained in March 2007 with pain and swelling of bilateral wrists, elevation of the inflammatory markers such as C-reactive protein (CRP), erythrocyte sedimentation rate (ESR) and matrix metalloproteinase-3 (MMP3). The blood examination showed anti-agalactosyl IgG antibody CARF positive, which is known as the early diagnosis marker for RA (Das et al. 2004). X-ray imaging showed particular bone erosions in the carpal bones of the hands and partially ankylosis of the carpal bones (Figure 1). RA diagnosis was met with the 1987 Rheumatoid Arthritis Classification. The combination of MTX (6–8 mg/week) and PSL (3 mg/day) was selected as an initial therapy for RA in September 2007. After that, however, disease activity was increased up to DAS28-CRP 5.1 (over right fingers, bilateral knees, right index middle ring PIP, and right ankle) and x-ray showed rapid destructive joints and high disease activity progression to Steinbrocker stage classification 3 within half a year after RA diagnosis. Then, she was referred to Takatsuki Red Cross Hospital for administration of anti-TNF-α inhibitor. Screening test was examined by included laboratory data with ECG, chest x-ray for excluding tuberculosis (Tbe), hepatitis C viruses (HCV) and hepatitis B viruses (HBV). Immunological examinations were tested for rheumatoid factor (RF) 22 U/ml, MMP-3 654 ng/ml, CRP 7.65 mg/dl, and ESR 35 mm/h. In August 2008, ADA (40 mg every other week) was administrated with MTX (8 mg/week). Seven days after receiving first administration of ADA, she showed rapid progression of sore throat, high fever and general lymph nodes swelling. Laboratory tests showed an elevated white blood cell count (WBC) 31800 (20.6% neutrophils and 63.7% lymphocytes without atypical lymphocytes), high level of CRP (3.3 mg/dl) and elevated liver-function tests with alanine aminotransferase level (53 U/L), aspartate aminotransferase (65 U/L), and gamma-glutamyltransferase (138 U/L). Evaluation of diagnosis with computed tomography (CT) and ultrasonography showed cervical and mediastinal lymph node swelling, multiple nodules measuring up to 13 mm in diameter in the lungs and mild hepatosplenomegary (Figure 2). An inguinal lymph node biopsy staining with hematoxylin and eosin staining (H&E staining) showed proliferation of the lymphatic cells without tumor cell phenotype (Figure 3A) and immunohistological studies with this examined inguinal lymph node revealed the lympho-proliferative CD20 positive cells (Figure 3B). H&E staining in high power filed showed the full range of mature lymphatic cells with polymorphous pattern and phagocytic macrophages included condensed small nuclear cells suggested apoptotic cell (Figure 3C). Besides, many lymphatic cells in the inguinal lymph node were positive with cleaved caspase 3 staining (Figure 3D). Moreover, EBV RNA *in situ* hybridization with the biopsy specimen revealed positive signals in the nucleus of large cells, and EBV DNA was detected by southern blot analysis (data not shown). Flow-cytometric analysis of infiltrating lymphocytes in lymph node showed an abundant population of CD38 positive B cell. In addition to routine blood test and urine cultures, the histories of infection were collected (e.g., HCV, HBV, tuberculosis, cytomegalovirus, parvovirus, varicella-zoster virus, HIV, EBV). The examinations for all these infections agents were negative except for high titers of anti-EBV antigen IgG (Table 1). Taken together these examinations, we diagnosed EB virus related B-cell LPD after the treatment with TNF-α inhibitor plus MTX, and that was in accordance with the 2008 WHO classification. Combination therapy with ADA plus MTX was terminated and pulse treatment with methylprednisolone was started. Steroid treatment for 7 days resulted in dramatic regression of LPD. Moreover, 10 days treatment with methylprednisolone reached to the complete remission of arthritis with below detection limits in ESR (3 ~ 11 mm/h), CRP (<0.3 mg/dl), and low level of MMP-3 (17.3 ~ 59.7 ng/ml) (Figure 4A and B). After the improvement of clinical symptoms and laboratory data, the administration of PSL was tapered off. She achieved drug-free with the reduction of EBV VCA-IgG titers (160 U/ml) 12 months after the emergent admission, and delivered healthy baby 2 years after discharge. She has completely attained drug-free Boolean remission for 5 years.

**Table 1:** **Immunological test on admission**

**Valuable**	**Value**
HBsAg	<1.0
HCV Ab	<1.0
HIV Ab	(−)
SIR-2R	6658
VCA-IgG	320
VCA-IgM	<10
EBV-EBNA	<10
EBV-LQ-T	5 × 10^6^
CMV-G/EI	25.9

HBsAg, hepatitis B surface antigen; HCV Ab, hepatitis C virus antibody; HIV Ab, human immunodeficiency virus antibody; SIR-2R, systemic inflammatory response-2 receptor; VCA-IgG, Epstein-Barr virus viral capsid antigen- Immunoglobulin G; VCA-IgM, Epstein-Barr virus viral capsid antigen- Immunoglobulin M; EBV-EBNA, Epstein-Barr virus nuclear antigen; EBV-LQ-T, Epstein-Barr virus LQTHIFAEV; CMV-G/EI, Cytomegalovirus antibody titer.

## Discussion and evaluation

We described the RA patient who achieved CR with receiving combined therapy of ADA together with MTX, and that was followed by the occurrence and steroid-dependent disappearance of immunodeficiency-associated LPD due to EBV infection. After that, RA patient has never experienced a recurrence without any medication for 5 years and delivered a healthy baby.

A recent study shows that the combination therapy with MTX and TNF-α inhibitor leads to better outcomes (Upchurch and Kay 2012). To prevent disease progression, treat-to-target recommendations for RA include the evaluation and adjustment of drug therapy at least every 3 months until a target level of remission or low disease activity is achieved (Emery et al. 2009). For the setting of the treatment goal, the identification of effective predictors associated with good clinical response has been investigated from both sides of clinical markers and biomarkers (van den Broek et al. 2013). Furthermore, from the risk-benefit point of view, discontinuing anti-TNF therapy after sustained remission has emerged among good-responders (Tanaka 2013).

Pathogenesis of our patient with EBV infection seems to be one of the possible candidates for CR in RA treatment. RA is characterized by defects in central B cell tolerance mechanisms and impaired T-cell responses to EBV antigens (Samuels et al. 2005, Lunemann et al. 2008). In fact, high titer of anti-EBV antibody was detected in our case, suggesting the involvement of the continuous EBV infection (Toussirot and Roudier 2008). Moreover, increased viral replication enhances EBV-specific immune responses in RA, which is followed by the increased frequencies of EBV-specific and IFN-γ-producing CD8+ T cells in peripheral blood with high level of viral genomes (Lunemann et al. 2008). Tan *et al.* have reported that the virus-specific CD8+ T cells within the joint showed activated and differentiated phenotype and played a ‘bystander’ role in the maintenance of inflammation in RA patients (Tan et al. 2000). These immune dysfunctions might be related with the mechanism of RA pathogenesis and the increased risk of lymphomas under immunosuppressive therapy. Moreover, previous study showed that lymphomas are often observed in EBV-positive patients with RA (Samuels et al. 2005). In our case, the serum examination before administrating of TNF-α inhibitors showed high titers of anti-EBV antigen IgG, and lymph node biopsy after administrating of TNF-α inhibitors revealed EBV derived LPD. This case report might suggest a possible predictor for good response and CR with anti-TNF therapy.

Subsequent mechanistic studies have addressed the involvement of TNF-α antagonists in TNF induced apoptosis. Latest data suggest that reverse signaling can be induced by TNF-α antagonists to induce the cytokine suppression and apoptosis via ligation of cell surface transmembrane TNF (tmTNF) (Wong et al. 2008). Actually ADA has high potential to induce apoptosis by the binding to tmTNF-α with its high affinity (Shen et al. 2006), and Infliximab affects EBV-positive B cell survival due to the increased expression of TNF-α (Baran-Marszak et al. 2006) suggests that the administration of TNF-α inhibitors might induce apoptosis in EBV infected B cells due to the high production of TNF-α in latent infected lymph node. In our case, we consider the possibility that the anti-TNF inhibitor induces apoptosis in LPD that leads to complete remission. The occurrence of LPD in RA patient with receiving immunosuppressive therapy is recognized as the “Immunodeficiency-associated LPDs” by the World Health Organization (WHO) and a possible link between TNF-α inhibitors treatment and increased risk of lymphoma has been suggested (Das et al. 2004). Taken together it is suggested that the administration of ADA plus MTX caused the driving re-activation of the EBV, and leads to EBV-associated LPD under immune-suppressive status. Increased awareness is necessary to monitor the possible occurrence of LPD before and after starting immune suppressive therapy.

## Conclusions

In this case, we experienced the remission of LPD after CR with combined therapy with ADA and MTX. RA is a polygenetic disease, it makes difficult to unveil the pathogenesis of RA and treatment. More evidence and further investigations are required for elucidating the mechanism of complete remission and establishing optimal treatment strategies.

## Abbreviations

RA: Rheumatoid arthritis
CR: Complete remission
TNF-α: Tumor necrosis factor alpha inhibitor
ADA: Adalimumab
MTX: Methotrexate
EBV: Epstain-Barr virus
LPD: Lymphoproliferative disorder
PSL: Prednisolone
CR: Complete remission
CRP: C-reactive protein
ESR: Erythrocyte sedimentation rate
MMP3: Matrix metalloproteinase-3
Tbe: Tuberculosis
HCV: Hepatitis C viruses
HBV: Hepatitis B viruses
RF: Rheumatoid factor
WBC: White blood cell
H&E staining: Hematoxylin and eosin staining
CT: Computed tomography
tmTNF: Transmembrane TNF
WHO: World Health Organization
